# The origins of the great pandemic

**DOI:** 10.1093/emph/eoz001

**Published:** 2019-01-21

**Authors:** Michael Worobey, Jim Cox, Douglas Gill

**Affiliations:** 1 Department of Ecology and Evolutionary Biology, University of Arizona, Tucson, AZ, USA; 2 Sydenham House, Applethwaite, Keswick, UK; 3 6 Northolme Road, London, UK

**Keywords:** influenza, 1918, pandemic, geography, origin

## Abstract

The timing and location of the first cases of the 1918 influenza pandemic are still controversial, a century after the pandemic became widely recognized. Here, we critically review competing hypotheses on the timing and geographical origin of this important outbreak and provide new historical insights into debates within military circles as to the nature of putative pre-1918 influenza activity. We also synthesize current knowledge about why the 1918 pandemic was so intense in young adults. Although it is still not clear precisely when and where the outbreak began and symptom-based reports are unlikely to reveal the answer, indirect methods including phylogenetics provide important clues, and we consider whether intense influenza activity as far back as 1915 in the USA may have been caused by viral strains closely related to the 1918 one.

The influenza pandemic of 1918 was arguably the most intense outbreak of infectious disease in human history. It killed an estimated 50 million people worldwide, most of them within a period of just a few months during the autumn of that year [1] (Fig. 1). In the intervening century, a great deal has been learned about the nature of this pandemic, not least that it was viral in origin, a fact not accessible to those who were caught in the midst of the outbreak. With the benefit of 100 years of research, we now know that the pandemic was caused by an influenza A virus (IAV) of the H1N1 subtype, so named based on antigenically distinct haemagglutinin (HA) and neuraminidase (NA) glycoproteins found on the exterior of the virion. To date, 18 different HA subtypes and 11 NA ones have been identified in animal (especially avian) reservoirs, and pandemics are characterized by cross-species transmission into humans of new or at least antigenically divergent HA and/or NA variants. The question of when, where and how the eight genomic segments that code for HA, NA and the other proteins of the virus entered the human population and caused such devastation—particularly amongst young adults around 30 years of age—is still an active area of research.

**Figure 1.**
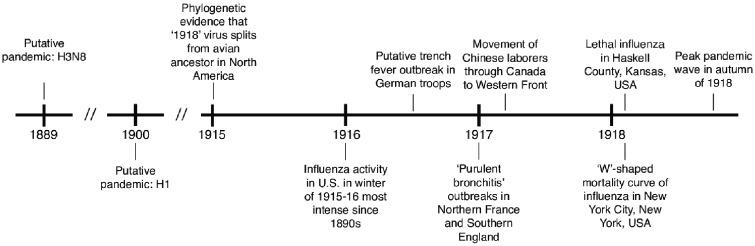
Some key events in the history of influenza that are discussed in this article

As it worked its way across the globe, the influenza virus of 1918 gave little warning to its victims that a visitation was at hand. One day, their communities were free from the affliction; on the next, their lives were dramatically transformed. The story was very often the same.

Again and again a perfectly healthy man may be taken ill in the street or on duty with a sense of general malaise…and rapidly develops such a sense of prostration that wherever he is he has to lie down… He gets to bed and is only too glad to stay there [2].

For the majority of victims, succumbing to the virus meant a simple case of ‘flu’. Recovery was ‘fairly speedy… and without sequelae’ [2]. No treatment was found to be of value in preventing or aborting an attack; but, by the same token, none could ward off, in a minority of cases, its development into a more virulent form of the disease. In these latter cases, the complications, initially pulmonary, broadened out and became systemic. The symptoms could take a variety of forms, but physicians laid stress on a single feature which, in the fatal or near-fatal cases, was common. For if, to produce the exact tint upon the patient’s face, ‘one would need to mix some heliotrope, or lavender, or mauvy-blue with red paint… the prospect is grave indeed’ [2].

Those who have chronicled the march of this pandemic have pointed to two distinct phases through which, during the year 1918, its incidence had moved. The first phase, which began in spring and lasted until August, was characterized by an acute onset and high fever. The illness was of short duration and fatalities were relatively few. Then followed, from the autumn onwards, a second and more destructive phase. It affected all ages and conditions, but as noted above its effects on young adults were particularly marked.

In recent years, the question has arisen as to when and where the first case of the influenza pandemic of 1918 may have been detected. At least three locations have been mooted. First, the historian John Barry has suggested that Loring Miner, a physician in rural Kansas, in the USA, encountered cases in the early weeks of 1918 which, while akin to influenza, posed an unusual risk to life [3]. Miner based his diagnosis on the symptomatology involved, and, interestingly, those symptoms did not include heliotrope cyanosis, which, as time drew on, came to be regarded as the tell-tale most closely associated with the pathogen involved, probably because some victims were asphyxiated by pus blocking their airways. Influenza was not a notifiable disease, but Miner, moved by the morbidity involved, sent off a report to the public health authorities [4]. The significance of the incident, in Barry’s view, lies in its timing and location. These deaths in rural Kansas took place in an area only a few hundred miles from a US Army Camp where, a few weeks later, Barry reports, one of the first recorded outbreaks of the pandemic may be said to have occurred.

A second location, from which the influenza outbreak may first have been reported, is to be found in northern France. Lt J.A.B. Hammond and two colleagues encountered an outbreak of ‘purulent bronchitis’ in late 1916 and early 1917 at a hospital centre forming part of the British army encampment at Etaples. An initial paper was published by this group in *The Lancet* in July 1917 [5]. This publication precipitated a rapid follow-up article, also in *The Lancet,* from an independent military group reporting similar observations at Aldershot, in the south of England [6]. Looking back 2 years later, this second group had no doubt that in 1916–17 they had observed ‘fundamentally the same condition as the “influenzal pneumonia” of this present [1918] pandemic’ [2]. The possibility that these outbreaks were caused by the very pathogen which soon thereafter killed 50 million people was contemporaneously put forward by American authorities [7]; and it has been made again, in recent years, by the virologist John Oxford [8].

The third claim to have located the first cases of the pandemic comes from those who follow the history of disease in China. In the course of several winters, concluding with that of 1917–18, epidemics that centred on afflictions of the lungs were seen in northern China [9]. In November 1918, during the main wave of the pandemic, voices were raised which pointed to these outbreaks in China providing a possible first case [10]. This hypothesis has been aired again, and a further argument adduced in its support: in that, from February 1917 onwards, some tens of thousands of Chinese volunteers crossed Canada on their way eastwards towards France, and thus perhaps brought the virus into North America and later Europe [9].

These three hypotheses have been launched by different groups of people looking back and reflecting on outbreaks of influenza-like disease that had been reported to the medical press prior to the Great Pandemic. Their reflections led them to build arguments for a relationship or cause. Of necessity, such hypotheses were based on symptomatology, and not always on much else. The science of microbiology, at the time of the Great War, had not yet reached a level at which a diagnosis of influenza could correctly have been made. (Indeed, as is well known, a mistaken link between ‘Pfeiffer’s bacillus’, a bacterium now called *Haemophilus influenzae,* and influenza, made by the bacteriologist Richard Pfeiffer, inadvertently caused much time to be wasted in profitless research.) Nevertheless the technique of re-reading the medical press with the aim of unearthing outbreaks of disease, which can then be linked to the pandemic, may yet be fruitful.

In this context, debates within both the German and the British armies as to the causes of mysterious ailments from which, in the months and years which led up to the pandemic, their respective troops had suffered, have perhaps not been properly explored. In our attempts to do so we found that in November 1916, in the pages of the *Feldärztliche Beilage*, a fortnightly supplement to the weekly *Münchener Medizinische Wochenschrift* (perhaps the most influential German-language journal at the time), a Dr Becher reported that, throughout that summer, the German army had had to cope with multiple soldiers with diarrhea and long-lasting fever [11]. Examination of their blood and feces had revealed nothing by way of a pathogenic agent. Accordingly, he and his colleagues had been hard put to classify the illness. Insofar as typhoid fever was not present, they had found it simplest to write of ‘influenza’. But Becher was not sure. For himself, he preferred to classify the ailment as ‘ein reines Influenzafieber ohne katarrhalische Erscheinungen mit Allgemeinsymptomen, wie Kopfschmerzen, Appetitlosigkeit’—a pure influenza fever without catarrhal symptoms and with general symptoms, such as headache and loss of appetite. And, in a passage which echoes the many words penned during that same month, of November 1916, by Sir William Leishman, Adviser in Pathology to the British troops in France—as he, Leishman, fought verbal battles with his colleagues, in and around Etaples, who had sought to classify all fevers of unknown origin as suspected typhoid or paratyphoid—Becher went on to discuss the difficulties faced by his own colleagues in distinguishing between typhoid fever and influenza [11].

In fact, an immense amount of effort appears to have been put in, by bacteriologists in both the German and the British armies, during 1916 and 1917, to pin down the real nature of the influenza-like illnesses with which the physicians in the military hospitals were faced. Leishman reports on his examination of copious case histories, from both the bacteriological and clinical perspective, during his tours of the dozens of hospitals with which he was involved [12]; and he mentions the drawing up of tables via which he could debate with colleagues the nature of the unknown ailment(s) with which they grappled at the time [12]. Still, little of Leishman’s paperwork, other than the extensive overview contained within his diary [12], appears to have survived, and it is not clear how many of the cases he investigated may have been influenza. And though the authors of the present paper are not intimately familiar with the records of the German army, it seems unlikely that the casenotes gathered by Becher and his colleagues can be called up and perused. At any rate, with the benefit of hindsight, the fever cases considered by Becher seem to us more likely to represent trench fever—a bacterial disease vectored by lice. We can be yet more certain that, apart from the few words penned by Loring Miner for the US *Public Health Reports*, nothing of that physician’s *oeuvre* survives to be discussed.

It is all the more remarkable, therefore, that, in the case of the second of the three hypotheses relating to the first reported case of the Great Pandemic, some of the raw material survives and can be re-examined and reviewed. At Etaples, in their pursuit of the ‘unusually fatal disease’, which they termed ‘purulent bronchitis’, Dr Hammond, Dr Rolland and Dr Shore conducted 156 consecutive autopsies during February and March 1917 [5]. Going further, they chose twenty typical cases, wherein the patient showed entirely characteristic symptoms, and they conducted tests upon the sputum [5]. Unwittingly, the three authors have left us a good deal more than a general list of symptoms and a table of the bacteria involved. The army records relating to the men dying in the Etaples hospitals have survived in perfect order [13]. So the precise dating by Hammond *et al.* of when the autopsy work was carried out has enabled us to identify all of the dead men, and to locate many of their medical histories, life stories, and the like. And something yet more precious has survived intact as well. A few microscope slides prepared by the pathologist Dr Rolland on the 1917 study [5] survive as family heirlooms, and we plan to test the hypothesis that these men suffered from a virus related to the 1918 one by screening them for viral RNA (Fig. 2). The slides are labelled with a name and date, and an indication of the type of organ tissue they contain.

**Figure 2.**
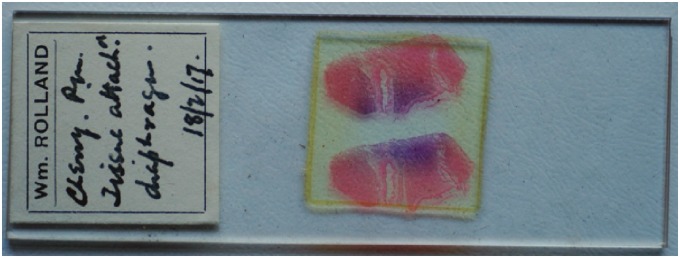
Slide from Dr William Rolland’s collection, from one of the cases documented in reference 3: Private Cherry, ‘tissue attached diaphragm’ 18 February 1917. No 41234 Private Ernest Cherry, 17^th^ Battalion, Highland Light Infantry, had died in No. 24 General Hospital, Etaples, on 8 February 1917 [13]

Setting aside, though, the extensive medical records underpinning Hammond *et al.*’s contribution to *The Lancet*, and ignoring, for the moment, the contents of Dr Rolland’s slides, another question nonetheless arises: what of the symptomatology involved?

Re-reading this list of ‘clinical features’, in the year 2018, and bearing in mind that, just a year thereafter, the influenza pandemic of 1918–19 began to take its toll, one of the five features stands out clearly from the page. That feature was cyanosis. It was referred to frequently, as the article progressed:

Dyspnoea and cyanosis are prominent features….Cyanosis is another prominent feature throughout the illness….[O]nce the patient ceases to bring up the purulent secretion he quickly goes downhill, becoming more and more cyanosed with right-sided failure of the heart….The face is more often than not cyanosed…,[T]he organs are congested, the heart dilated, though fairly healthy, and the patient cyanosed… [5].

Of note is that the right-sided heart failure mentioned here is consistent with mucus congestion in respiratory tissues, which are fed by the right ventricle of the heart. The human body’s reflex response to obstruction of the upper airways by thick, sticky mucus and pus is, in effect, to ‘try harder’. Increased respiratory effort reduces pressure within the thorax, which in turn leads to increased return of venous blood from the body to the right ventricle and right-sided heart failure (Because the heart and lungs are both within the thorax, return of venous blood from the lungs to the left ventricle is not increased.). The bacteriology of the twenty sputum cases is interesting as well. The three physicians found the bacteria characteristic of bronchitis, pneumonia, and so on, and, in many of the twenty cases, Pfeiffer’s bacillus too. At no stage, though, did they generalize about the role played by the latter, or comment more generally on the role of influenza in the ‘minor epidemic’ which they faced.

Although reconstructing detailed histories on all the cases reported by Hammond *et al.* is beyond the scope of the current work, analysis of the first 6 in the series of 156 consecutive disease-related deaths does reveal mention of influenza, along with multiple cases of serious lung infections typical of sequelae to influenza infection [13]:

Case 1, No. 44780 Sapper Alfred Elmore, Royal Engineers. Medical record destroyed: but he died of broncho-pneumonia on 01 February 1917.

Case 2, No. 2607 Private Henry Hague, Australian Imperial Force. Medical record reveals diagnoses as follows:

-Admitted to an Etaples Hospital with pleurisy 27 January
-‘Died of pneumonia’ 1 February

Case 3, No. 9635 Private John Hill, Royal Sussex Regiment. Medical record destroyed: but he died of broncho-pneumonia on 01 February.

Case 4, No. 53553 Lance Corporal George Godwin, Royal Fusiliers. Medical record destroyed; and nothing known of his cause of death on 1 February.

Case 5, No. 24954 Private Henry Wilkinson, Kings Own Regiment. Medical record reveals diagnoses as follows:

-Field Ambulance diagnosis, 28 January: ‘Bronchitis’
-Casualty Clearing Station diagnosis, 29 January: ‘Influenza’
-Etaples Hospital, cause of death, 2 February: ‘Nephritis’.

Case 6, No. 22993 Private John Vernon, King’s Own Regiment. Medical record reveals diagnoses as follows:

-Etaples Hospital diagnosis, 28 January: ‘Not Yet Diagnosed’
-Etaples Hospital cause of death, 2 February: ‘Acute Nephritis’.

Hammond *et al.* summarize the results of their work in the following words: ‘The disease has been very fatal. This is shown most readily by post-mortem records referring to this period. During February and early March, while the outbreak was at its height 45% of the necropsies in this area showed the presence of purulent bronchitis’ [5].

One additional word in relation to what had happened at Etaples: it was not a remote spot at which three physicians, working in isolated fashion, encountered and wrote up an affliction which rose up like a bubble and then died. Its 15 or so hospitals gathered in some 20 000 thousand beds—the largest-ever overseas concentration of hospitals in the British army and probably in any army of that day. There were thousands of physicians, surgeons and nurses working at the Base. It so happened, though, that two pathologists and a bacteriologist alert in mind and highly motivated too, applied themselves to the solution of a problem which others faced in a competent but less ambitious way.

Even so, those other professionals, who may or may not have known that Hammond and his colleagues were writing up the case, have contributed to our understanding of what was taking place. Take, for instance, the case of Private Walter Scott, ‘a well-developed, well-nourished male of about 33 years of age’. A native of the north of England, he served only for the briefest time in France before being brought as a patient to No. 1 Canadian General Hospital, Etaples, suffering from no more than a ‘chilly sensation, headache, general pains, dry cough and slight sore throat’. Four weeks later, on 14 February 1917, the writing on the soldier’s record card was rather less benign. ‘Severe cyanosis. Whole of right lung shows signs of consolidation. Sharp crackles through left lung’. Scott’s case was unusual in that, when he died, the phrase ‘Disease influenza, complication lobar pneumonia’, was written as the cause. Seemingly, this led the authorities in London to ask for a more comprehensive account of what had taken place [14]. Such questioning was rare: but it arose, presumably, because senior London-based physicians could not understand how an ailment like influenza could possibly have killed a healthy young adult. Incidentally, the examination *post-mortem* of Private Scott appears to have formed the 55th, out of the 156 consecutive autopsies, upon whose findings the Hammond *Lancet* article was built. Similarly, in the case of the 17th such death, a senior physician, not part of *The Lancet* threesome, felt impelled to write up the history of the case. Lieut. Col. SA Owen, Royal Army Medical Corps, wrote of the patient becoming ‘much more cyanosed’, as the days went on, and confirmed that ‘P.M. Examination showed Purulent Bronchitis’ [15].

Sir William Leishman, Advisor in Pathology to the British Expeditionary Force, provides one additional clue that supports the idea that the ‘purulent bronchitis’ observed in Etaples, (and Aldershot) was linked to influenza. Leishman had probably encountered Dr Hammond and Dr Rolland during their service in France, but he was in no way connected with the research they did at Etaples. As it happens, though, Leishman reports on a quite separate link between influenza and a fatal form of bronchitis during the very days when Hammond et al were driving through their programme of ‘156 consecutive necropsies’.

In the relevant extract from Leishman’s War Diary [12] Leishman is sitting in his office in Abbeville, not far from the coast, and is replying to correspondence emanating from a Colonel Soltau, whose responsibilities lay right at the Front:

Abbeville, 12 February 1917. Replied to a letter from Col. Soltau, the new Medical Consultant of the 1^st^ and 2^nd^ Armies who told me he had seen recently some very severe and rapidly fatal cases of Bronchitis, which he thought were Influenza in type, and asking me whether there was a stock Influenza Vaccine which might be expected to do any good.

Our view is that attempts to pin down the exact location of the first cases of any influenza pandemic are fraught with difficulties, and, for at least two of the three hypotheses noted above there exists strong contrary evidence. The simplest objection is to the idea that Chinese labourers were responsible for the spread of the pandemic virus. Crucially, Shanks [16] showed that influenza cases among Chinese and Southeast Asian labourers and military recruits lagged, rather than led, cases among other groups in the same locales. Moreover, Chinese labourers were also shipped to Europe from the East via Suez or the Cape, although these routes were rapidly abandoned in March of 1917 in favour of transporting them across Canada [10]. Finally, although the Chinese workers were reportedly transported across Canada in sealed trains, it seems unlikely even under this harsh scenario that the virus would not have initiated detectable spread in Canada if these individuals really were the original hosts of the pathogen.

More subtle is the evidence against an origin of the pandemic in Haskell County, Kansas in early 1918. This hypothesis is challenged by epidemiological data from 1917/18 influenza activity in New York City in a 2005 paper by Olson *et al.* [17]. Evidently, the virus circulating in the spring of 1918 in NYC already was targeting young adults, giving rise to the so-called ‘W’-shaped mortality curve often commented upon in the fall 1918 wave (with mortality among young adults giving rise to a middle peak, rather than the expected ‘U’-shaped curve with deaths primarily in the young and the old). This is important because it suggests that cases of the virus were already circulating in other parts of the USA by early 1918 (and in sufficient numbers to be detected in routinely collected public data, which is remarkable). The Kansas cases no doubt make for a compelling story, but one that has perhaps left a stronger-than-deserved impression that they were the very first cases. Moreover, the noteworthy influenza outbreak at Camp Funston, Kansas, in March 2018, was mild, with many cases but few deaths. This was after New York City already had evidence of very high influenza mortality (higher than any other period except for the fall of 1918, in fact) and increased mortality in young adults [17]. The virus causing those cases in New York City in February presumably predated the March outbreak at Camp Funston. So what happened on this army base in Kansas was not the first large outbreak of the 1918 pandemic. Either way, these cases in Kansas and New York suggest that a highly virulent virus was already circulating by early 1918 at the latest. This epidemiological record mirrors that of viral sequences from influenza victims in the spring of 1918, which show no obvious differences from cases during the intense autumn wave that could explain a general difference in virulence of the various waves of the pandemic [18]. Whether these possible early manifestations of the 1918 pandemic virus were co-circulating with genetically distinct, less-virulent IAV strains that caused milder cases during the same period remains to be determined.

Although the even earlier cases noted in France and the UK are compelling, we contend that early documentation of plausible cases is highly unlikely to indicate geographic origin. In other words, even if there were early cases in France and the UK in 1916 and 1917, this still would not necessarily indicate that the pandemic virus first arose in Western Europe. To our knowledge, the only reasonably persuasive evidence we have of geographical origin, which is by no means conclusive, comes from phylogenetic analyses indicating that most of the avian-like genomic segments in the 1918 human virus appear to be of Western Hemisphere and, probably, North American origin [19] and that the pandemic virus’s HA gene was likely circulating in the human population for many years prior to 1918 [20]. These studies suggest, moreover, that the virus reassortment event giving rise to the pandemic probably occurred in or around 1915—since the common ancestor of human and swine H1N1 genomic segments, and in some cases the common ancestor of human, swine, and avian segments, can be dated to that time window—long before the Kansas cases. Therefore, we should remain cautious in the face of incomplete knowledge. In a nutshell, the chance that a very precise epicentre of the pandemic was captured and publicly documented in real time by front-line observers, whether in Kansas, Etaples, China or wherever, must be close to zero. On the other hand, the chance that early documented cases will be proffered at some later point as the first cases is clearly very high: such is the nature of headline-making pandemics.

Given the possibility that the 1916–17 cases in Etaples and Aldershot were early manifestations of the ‘1918’ virus, and in light of the phylogenetic indications that the virus may indeed have had its genesis a few years before 1918, it is worth pointing out that 1915–16 Northern Hemisphere influenza was particularly intense in the USA. A *Public Health Reports* article in early January 1916 [21] noted that, ‘During the last few weeks what is reported to be influenza has become epidemic in practically all parts of the United States. It is present from the Atlantic seaboard to the Pacific coast and has spread even to such regions as central New Mexico’. Some reports from individual locales sound remarkably similar to reports during the peak of pandemic activity in the fall of 1918. For example, in Philadelphia

Senior Surg. Irwin reported December 31, 1915: Influenza epidemic here, total deaths five weeks ended today 141, 72 deaths last week…total cases of pneumonia in December 881, total deaths pneumonia last week 284…. a great many of the various services of the city have been badly crippled by the number of cases of illness.

And, in Chicago, ‘Surg. Cobb reported December 31, 1915: Influenza not a reportable disease. This week so far reported 57 deaths from influenza, last week 30 deaths. Fourteen hundred and forty cases pneumonia reported this month, 666 deaths, 201 deaths within the last four days.’ Reflecting, at a distance of some months, on the ‘epidemic of grip’ which had ‘swept over’ the USA, the authors of an article in the *Journal of the American Medical Association* summed up the consensus: ‘The older practitioners can recall no similar epidemic during the twenty-five years intervening between 1890 and this year’ [22]. These reports, along with the phylogeographic evidence noted above, point to the possibility of a surprisingly early North American origin of the ‘1918’ pandemic.

Because the 1889 pandemic unfolded with three major waves worldwide from 1889 to 1892 [23], with the 1892 wave being the most intense, at least on the East Coast of the USA, it seems worth considering whether so-called herald waves of the 1918 pandemic might have stretched back all the way to 1915. Viruses with identical or nearly identical genome sequences to those in the fall wave were circulating in early 1918 [18], at a time prior to the recognition of a new pandemic. It is perhaps not so great a leap to imagine that these viruses were related to those circulating the previous winter in Etaples, and the winter before that in the USA, and that the intervening Northern Hemisphere summers played a role in temporarily dampening the outbreak before demobilization at the end of World War I precipitated massive outbreaks worldwide. At any given time, two million men and women from the UK, Canada, Australia, New Zealand, China, India, the West Indies, South Africa, Fiji, Portugal and elsewhere were serving with the British army in the north of France. Repatriation of survivors at the end of hostilities likely contributed to rapid worldwide dissemination of the deadly virus and the initiation of its most intense wave.

Though the exact timing and location of the first cases of the pandemic remain obscure, notable progress has been made in the century since 1918 on why the pandemic affected young adults so intensely, and on how this connects the 1918 experience with later pandemics. Crucially, antigenic imprinting (also known as original antigenic sin) caused by an individual’s first IAV exposure(s) in infancy to one or the other of the two HA phylogenetic ‘groups’ (Fig. 3a) has emerged as a key explanatory factor that appears to underlie not only the peak in mortality among young adults in 1918, but also the troughs in mortality in those who were slightly younger or slightly older (Fig. 3b) [20]. Accordingly, the year of birth of victims and survivors of the 1918 pandemic has been revealed to be a powerful predictor of severity of disease [20], because one’s year of birth reflects the first IAV strain to which one was exposed in infancy. For the young adults who suffered the highest mortality rates in 1918, the sharp peak in death rate from the 1918 influenza virus closely recapitulates the exposure of that same cohort to a putative H3N8 pandemic virus that emerged in 1889 and may have been replaced by an H1 strain around 1900 [20, 24] (Fig. 3). This cohort, centred on those who were 28 years of age in 1918, were exposed in childhood to a group 2 HA (i.e. H3) that was mismatched to the group 1 HA (H1) of the 1918 pandemic virus. The majority of those slightly older and slightly younger may have experienced an initial childhood exposure to a group 1 HA and subsequently benefited from relatively good immunity to the pandemic virus [20, 25].

**Figure 3.**
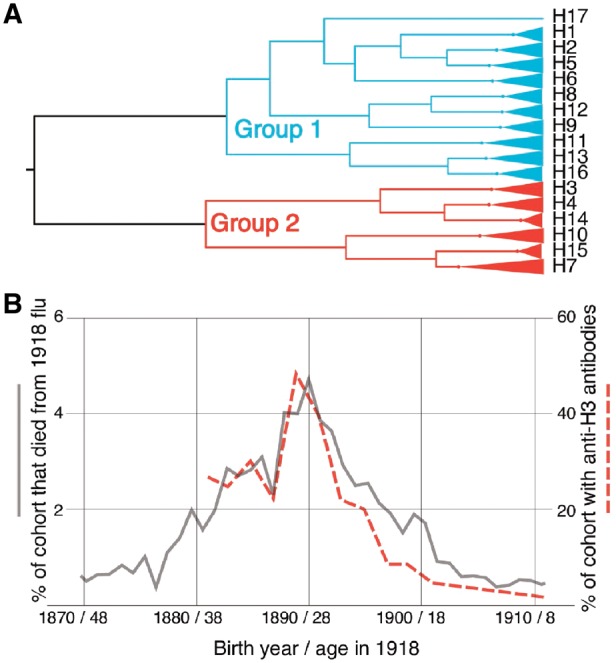
HA groups and relative pandemic death rates and relative proportion of each age group exposed to a mismatched, putative H3 HA in childhood. Panel (**A**) is adapted from Fig. 3 of reference 19. It depicts the two recognized phylogenetic groups on the HA evolutionary tree. Note that H3, the putative childhood virus of most young adults who died in the 1918 pandemic is in the opposing group relative to the 1918 H1N1 virus. Panel (**B**) is adapted from Fig. 1 of Worobey *et al.* [20] and references therein. The horizontal axis depicts cohorts of various birth years and ages in 1918. The dashed red line indicates the percentage of each birth year cohort showing strong reactivities to H3 antigens. The grey line shows the percentage of each age group that succumbed to the 1918 influenza virus

A similar pattern can be observed in ongoing cases of avian-origin infection by viruses including H5N1 and H7N9 [25]. As in 1918, when a generation of young adults exposed as children to a group 2 HA suffered severe outcomes when later infected by a group 1 HA virus, most cases of H5N1 (group 1) are observed in those born after 1968, exposed in infancy to the human group 2 H3N2 virus. Conversely, most cases of H7N9 (group 2) are observed in those born before 1968 and exposed as kids to either H1N1 or H2N2, both of which are group 1 viruses. Hence, as a general phenomenon, first exposure to an influenza virus appears to determine the group of viruses to which a person develops a life-long immunological imprint. Individuals who were born during a period when their first exposure to IAV was to a strain of the opposing phylogenetic HA group have immunologic imprinting for the ‘wrong’ group of viruses and are therefore at increased risk, possibly because they are less able to recall protective responses to conserved epitopes of the HA stalk domain, which tend to be shared within HA groups [20, 25].

The 1918 pandemic was caused by an H1N1 virus (with a Group 1 HA). Those whose first exposure had been to a putative H3N8 (Group 2) virus that emerged in 1889 were at high risk of death [20]. Crucially, because children born several years prior to a newly emerged IAV strain can experience that virus as their first (or among their first) IAV infections, one should not expect a clean demarcation of increased risk to coincide with the year of emergence of that H3N8 strain. Although the young-adult mortality rate from the 1918 virus has a sharp peak in those born very near 1889, it stretches back to include those born up to a decade or so prior to 1889 (Fig. 3b). Interestingly, it also stretches forward only a decade or so, possibly because a new H1 virus emerged in the early years of the 20th century, displacing the 1889 H3 virus [20, 24]. This idea is supported by the lack of evidence of anti-H3 antibodies in those born after the turn of the century—despite clear evidence of N8 reactivities until shortly before 1918—as well as by the low mortality during the 1968 H3N2 pandemic in those born before, but not after, about 1900 [20].

Although there is some evidence that the 1918 virus may have elicited unusually strong innate immune responses—a so-called ‘cytokine storm’ [26]— we believe the imprinting hypothesis explains the data better than the hypothesis that the young-adult mortality in 1918 was a consequence of that cohort having stronger immune systems, and hence suffering greater negative effects than other age groups. First, we see no reason, under the cytokine storm hypothesis, that the peak in mortality should have been centred, and sharply so (Fig. 3), on 28-year-olds. Do 28-year-olds really have markedly stronger innate immune responses than 18-year-olds? Second, the primate experiments suggesting unusual lethality of the 1918 virus included only a single modern control strain. Perhaps a larger null distribution of other H1N1 strains would show that the 1918 virus was not so unusual in its severity. Finally, because virtually all adults have had prior exposure to IAV, experiments on immunologically naïve animals [26, 27] may generate misleadingly severe outcomes: if the same animals had been previously infected by IAV at a young age, then secondarily exposed to the 1918 virus as adults, it is unlikely they would have experienced such severe symptoms. Nevertheless, results indicating that the 1918 virus is unusually lethal in mice [27], along with the fact that introduction of all new internal proteins in the 1918 virus could have played some role in its unusual virulence (due, e.g., to absent cellular immunity to new T cell epitopes) [20], suggest that the overall virulence of the 1918 virus may have been affected by factors other than antigenic imprinting in childhood. These uncertainties make the recovery of archival viral strains from prior to 1918 particularly attractive: exposing experimental animals such as ferrets, pigs or mice to reconstructed versions of putative H3N8 and H1N8 viruses that may have provided distinct imprinting of different cohorts in 1918 [20] may be the only way to resolve these questions and answer, finally, why this pandemic was so catastrophic.

Returning to the 1917 paper by Hammond, Rolland and Shore [5]: by describing a ‘symptom complex so distinctive as to constitute a definite clinical entity’ they provided what may be the first clear account of the pandemic disease that peaked in 1918 and became known as ‘Spanish flu’. With great clarity the authors described the clinical features and rapid progress of the disease, which typically resulted in death by asphyxiation caused by pus-filled airways. They described their unsuccessful attempts at treatment and alluded to their efforts to produce a vaccine. As medical officers so close to the front in the British army’s biggest ever overseas military hospital camp, their priority must have been to attend to the thousands of soldiers with wounds. Nevertheless they recognized an unexplained disease outbreak as a scientific challenge that they should study, record and report. The paper is thus of historical importance and a credit to the authors and to *The Lancet* who saw fit to publish it. Astute observations may have allowed these researchers to detect early embers of one of humanity’s great calamities even in the midst of another. More than a century later, their work, together with that of the others who provided early clues about the 1918 pandemic, deserves commemoration.

**Conflict of interest:** None declared.

## Funding

Michael Worobey is supported by the David and Lucile Packard Foundation. Jim Cox and Douglas Gill have no funding to report.
